# Postural stability in people with visual impairment

**DOI:** 10.1002/brb3.1436

**Published:** 2019-10-02

**Authors:** Ahmad H. Alghadir, Abdullah Z. Alotaibi, Zaheen A. Iqbal

**Affiliations:** ^1^ Rehabilitation Research Chair College of Applied Medical Sciences King Saud University Riyadh Saudi Arabia; ^2^ Department of Optometry College of Applied Medical Sciences King Saud University Riyadh Saudi Arabia

**Keywords:** COG velocity, postural stability, visual impairment

## Abstract

**Background:**

The visual system enables the brain to assess information regarding the position of the body in space. Congenital or acquired blindness leads to the development of abnormal sensory‐motor interactions that results in development of typical musculoskeletal deformities and gait patterns that cause disability. Diabetes and related complications are expected to increase exponentially in the next 10 years; thus, the number of people with visual impairment is expected to increase. However, there have been few studies regarding etio‐pathogenesis of postural alteration and balance impairment in people with visual impairment; moreover, no previous study has investigated postural stability in this population. This study aimed to assess the center of gravity (COG) velocity in subjects with visual impairment and compared with that in sighted subjects.

**Methods:**

Seventy male subjects, 20–40 years of age, participated in this study; they were divided into sighted (control) and visually impaired groups. COG velocity while standing on an unstable surface was measured using the NeuroCom^®^ Balance Master Version 8.5.0. For the sighted group, data were recorded with eyes open and then with eyes closed. For the visually impaired group, no instructions were given with respect to eyes during data collection.

**Results:**

Mean COG velocity was significantly higher in the visually impaired group than in the sighted group with eyes open. However, there was no difference in mean COG velocity between the visually impaired group and the sighted group with eyes closed. The difference in mean COG velocity between sighted subjects with eyes open and eyes closed was also significant. Mean COG velocity while standing on a foam surface varied among visually impaired subjects and sighted subjects with eyes open and closed.

**Conclusion:**

This study showed that subjects with visual impairment, regardless of eye opening or closure, behave in the same manner as sighted subjects with eyes closed.

## Graphical abstract

This study was aimed to assess center of gravity (COG) velocity in people with visual impairment and compared with that measured in sighted subjects.

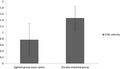

## 1 INTRODUCTION

Among all sensory systems of the body, humans primarily use vision to enable the brain to assess information regarding the relative position of the body in space and adjust the posture accordingly (Carroll, 1961; Roth, 2009; Stambough, Dolan, Werner, & Godfrey, 2007; Stones & Kozma, 1987). Visual acuity comprises the ability of the eye to perceive the shape of objects. People with visual impairment exhibit visual acuity of less than 6/60 in their better eye; visual acuity less than 3/60 in the better eye is referred to as blindness (Acheson, 2010; De Araújo et al., 2014). Congenital or acquired loss of vision leads to the development of abnormal sensory and motor interactions that result in the development of typical musculoskeletal deformities; these then lead to faulty gait patterns, which cause disability (Alotaibi, Alghadir, Iqbal, & Anwer, 2016; Barlow, 1955; Nakamura, 1997). People with visual impairment are unable to perform their daily activities, become dependent, and experience poor quality of life (Markowitz, 2006).

In addition to balance control, vision is necessary to provide information regarding the orientation of the body in space, as well as precision of movement and timing of motor reaction; notably, experience alone cannot compensate for these characteristics with respect to loss of vision (Alotaibi et al., 2016). The gross movements of blind people are poorly developed and delayed due to overprotection by parents and teachers during childhood (Buell, 1950). Visual impairment leads to increased social dependence, restricted mobility, increased anxiety, a persistent fear of falling, and increased likelihood of falling (Varma, Wu, Chong, Azen, & Hays, 2006; Tinetti, De Leon, Doucette, & Baker, 1994); these aspects often cause affected individuals to remain isolated from the external environment (Miller, 1969). Notably, their educational and social development is also severely affected (Dickinson & Leonard, 1967). Evaluation of the performance of such activities is important in the rehabilitation of people with visual impairment.

Postural stability is the ability of the body to maintain balance (Malheiros et al., 2013; Rajendran & Roy, 2011); it is often measured by postural sway, which constitutes continuous deviation and correction of the center of gravity (COG) on a relatively small base of support (MacLeod, 1996; Tabak et al., 2009). Visual feedback is a major factor that contributes to balance control, especially during standing (Grace Gaerlan, Alpert, Cross, Louis, & Kowalski, 2012; Palm, Strobel, Achatz, Luebken, & Friemert, 2009). Notably, COG sway increases and postural control is disturbed when eyes are closed in sighted subjects (Giagazoglou et al., 2009); moreover, increased postural sway increases the risk of falling (Hughes, Duncan, Rose, Chandler, & Studenski, 1996; Siriphorn, Chamonchant, & Boonyong, 2015).

Although several reports have been published regarding people with visual impairment (Al‐Salem & Rawashdeh, 1992; Awad, Al‐Eisa, & Alghwiri, 2014; Mansour et al., 1997; Tabbara, 2001; Tabbara & Badr, 1985), few of them have discussed the etio‐pathogenesis of postural alteration and balance impairment in this population (Campayo‐Piernas, Caballero, Barbado, & Reina, 2017; Kotb, Hammouda, & Tabbara, 2006); moreover, no previous study has investigated postural stability in people with visual impairment. This study was aimed to assess COG velocity in people with visual impairment and compared with that measured in sighted subjects. The findings of this study will fill a gap in the literature and further help to develop therapeutic intervention for postural and balance impairment in people with visual impairment. In addition, these findings will enhance public awareness for complete rehabilitation of this population.

## 2 METHODS

### 2.1 Subjects

Seventy male subjects, 20–40 years of age, participated in this study; they were divided into two groups, the visually impaired group and sighted (control) group. The visually impaired group comprised 35 subjects with visual acuity less than 3/60 in both eyes; all of these subjects were from a school for the blind (Bucci et al., 2009). The sighted group comprised 35 subjects with visual acuity at least 6/60 or better even after correction, matched for age with subjects in the visually impaired group. Subjects were excluded if they exhibited any signs or symptoms of cognitive or balance impairment, temporomandibular joint disorder, or any other skeletal anomaly or reported any history of balance training. All the subjects were informed of the aims and objectives of the study and written informed consent for participation.

### 2.2 Procedure

Center of gravity velocity was measured while subjects stood on an unstable surface standing with their hands by their sides using the NeuroCom^®^ Balance Master (version 8.5.0; Neurocom International Inc.; Alghadir, Zafar, & Iqbal, 2015; Chien, Hu, Tang, Sheu, & Hsieh, 2007; Liston & Brouwer, 1996; Newstead, Hinman, & Tomberlin, 2005). Subjects stood on a 50 × 50 × 15‐cm foam block (provided with the Balance Master), placed on a 46 × 152‐cm force platform; data were collected by a connected computer. The COG velocity of natural postural sway was measured as degrees per second (deg/s) and sampled at a frequency of 100 Hz. During data collection, the Balance Master calibrated automatically.

For the sighted group, data were recorded in two conditions: with eyes open and with eyes closed. For the visually impaired group, no instructions were given with respect to eye opening or closure during data collection. There were three 10‐s trials for each test, with a rest of approximately 60 s between each trial. The mean values for the three trials were used for statistical analysis.

### 2.3 Statistical analysis

Mean and standard deviation (*SD*) was used for descriptive statistics. The normality of the data distribution was assessed using the Kolmogorov–Smirnov test. Because the COG velocity between the visually impaired and sighted groups did not show a normal distribution, this comparison was tested using a 0.05 level of significance with the Kruskal–Wallis test (Nonparametric ANOVA). Graph‐Pad Instat 3.0 (GraphPad Software) software was used for analysis.

### 2.4 Ethics approval

This study met ethical standards for human research in accordance with the Declaration of Helsinki. It was approved by the Rehabilitation research review board for ethics (Ref no. KSU/RRC/050/03).

## 3 RESULTS

The mean ages of subjects in the visually impaired and sighted groups were 28.8 ± 6.86 years and 28.97 ± 6.57 years, respectively. There was no significant difference in age between the two groups (*p* > .05).

### 3.1 COG velocity in sighted and visually impaired groups

The mean COG velocity while standing on a foam surface varied among visually impaired and sighted groups with eyes open and closed. In the sighted group, the mean COG velocities were 0.77 ± 0.37 deg/s (eyes open) and 1.54 ± 0.66 deg/s (eyes closed). The mean COG velocity in the visually impaired group was 1.47 ± 0.52 deg/s.

### 3.2 Comparison of COG velocity in sighted and visually impaired groups

In the sighted group, the mean COG velocity was significantly higher with eyes closed than with eyes open (*p* < .001).

**Figure 1:**
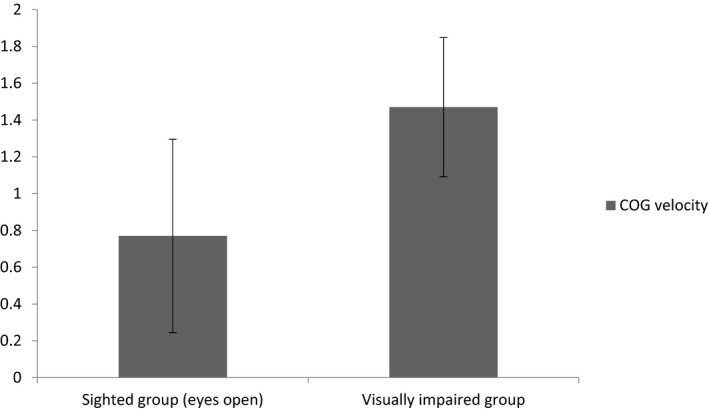
Mean and standard deviation of center of gravity (COG) velocity between sighted group (*n* = 35) with eyes open and visually impaired group (*n* = 35) while standing on a foam surface. Note the significant difference (*p* < .0001) between the two groups of subjects

In between‐group comparisons, mean COG velocity was significantly higher in the visually impaired group than in the sighted group with eyes open (*p* < .001; Figure 1). However, there was no difference between the mean COG velocity of the visually impaired group and that of the sighted group with eyes closed (*p* > .05).

## 4 DISCUSSION

The visual system is the primary sensory system that enables the body to assess various external factors and adjust posture accordingly (Carroll, 1961; Cascio, 2010; Roth, 2009). Postural stability is critical for maintenance of body balance (Barlow, 1959; De Araújo et al., 2014). When vision is lost, abnormal postural and motor patterns develop, leading to postural and balance deficits (Jeon & Cha, 2013; Ross, 1977). This study was performed to compare COG velocity between subjects with visual impairment and sighted subjects, in order to determine possible similarities or differences. To the best of our knowledge, this is the first study from Saudi Arabia and the surrounding region to investigate postural stability in people with visual impairment.

The results showed that mean COG velocity while standing on a foam surface varied among visually impaired and sighted subjects with eyes open and closed. The mean COG velocity was significantly higher in the visually impaired group than in the sighted group with eyes open. However, there was no difference in the mean COG velocity of the visually impaired group and the sighted group with eyes closed. Notably, the difference in mean COG velocity between sighted subjects with eyes open and eyes closed was significant.

When vision is lost, approximately half of the normal sensory input of the brain is lost (Stambough et al., 2007). Abnormal postural reflexes and motor patterns develop in blind people, which lead to an unusual distribution of muscular forces in the body that precipitates postural and balance deficits (Barlow, 1959; Jeon & Cha, 2013; Ross, 1977). Furthermore, “hand to eye” coordination is lost and hands must perform both perception and execution tasks (Miller, 1969). The loss of sensory feedback affects body mechanics, resulting in imbalance, loss of protective reflexes, and motor and neurological abnormalities (Joseph, 1964; Leonard & Newman, 1967). Vision does not affect the muscles' ability to exert force; however, neuro‐sensory motor development in blind people may contribute to balance disturbance (Giagazoglou et al., 2009; Juodžbalienė & Muckus, 2006). Such complex interactions may explain the significant reduction in postural stability in subjects with visual impairment, compared to sighted subjects as seen in this study.

The lack of difference in COG velocity between subjects with visual impairment and sighted subjects with eyes closed, as well as the significant difference in mean COG velocity between sighted subjects with eyes open and eyes closed, is consistent with the findings of previous studies that show that postural control decreases when eyes are closed (Hallemans et al., 2009a, 2009b; Palm et al., 2009; Sobry, Badin, Cernaianu, Agnani, & Toussaint, 2014). It has been well‐established that vision is the predominant input method used to maintain body posture, because of its high resolution regarding body position in space, compared to vestibular or proprioceptive inputs (Easton, Greene, DiZio, & Lackner, 1998; Grace Gaerlan et al., 2012). Notably, synchronicity among visual, somatosensory, auditory, and motor systems is attenuated when eyes are closed (Xu et al., 2014). Closing eyes has been shown to cause 20%–70% increase in postural sway (Lord, Clark, & Webster, 1991). Some studies have shown that blind subjects can maintain better equilibrium during static or dynamic postural tasks, compared to sighted subjects, whereas other studies have shown an inverse relationship (Juodžbalienė & Muckus, 2006; Portfors‐Yeomans & Riach, 1995; Pyykkö, Vesikivi, Ishizaki, Magnusson, & Juhola, 1991; Stones & Kozma, 1987). The results of the current study confirm that subjects with visual impairment, regardless of eye opening or closure, behave in the same manner as sighted subjects with eyes closed (Schmid, Nardone, De Nunzio, Schmid, & Schieppati, 2007).

A Little has been reported about differences in postural control among subjects with congenital and acquired blindness. Blind individuals adapt to the deficiency by developing mechanisms for postural adjustment (Schmid et al., 2007; Stones & Kozma, 1987). Timing of the lesion and rehabilitation program can stimulate compensatory mechanisms for postural control (Soares, 2011). Research has shown that environmental constraints affect pattern of postural response (Horak & Nashner, 1986). Congenitally blind children have a better capacity of combining the available nonvisual feedbacks, e.g., auditory feedback, and benefit from neuroplasticity of the unused visual cortex, as in reading Braille (Bavelier & Neville, 2002; De Volder et al., 1999; Held, Freedman, & Harris, 1996; Théoret, Merabet, & Pascual‐Leone, 2004). Neural connections can be modified with extensive use, practice, and training (Goldreich & Kanics, 2003; Kowalewski, Kattenstroth, Kalisch, & Dinse, 2012; Roland et al., 2013; Wong, Peters, & Goldreich, 2013).

The rehabilitation of a person with visually impairment should be initiated at an early stage, in order to facilitate their ability to adapt to the environment and achieve independence (Ribadi, Rider, & Toole, 1987). The posture adopted by a blind person due to natural compensation includes multiple deficiencies, none of which can be treated in isolation (Miller, 1969). The rehabilitation program for a person with visually impairment is a multidisciplinary task that should involve specialists from different medical fields (Alotaibi et al., 2016). Before restoring gait and posture, the missing link with the environment must be re‐established; this can be achieved by the use of a long cane (Miller, 1969), which substitutes some visual functions and prevents posture and gait disturbances (Siegel & Turner, 1965). When the link with the external world has been established, postural training should begin to improve mobility (Siegel, 1970). Training should be applied for the use of available sensory inputs (e.g., hearing to analyze orientation; Miller, 1969). Increased physical activity is also an important factor for refinement of nonvisual postural control systems (Nakata, Enzaki, & Yabe, 1994). A robust collaboration is needed among clinicians, teachers, and parents of children with visual impairment to formulate a rehabilitation program and motivate such children to follow the exercises appropriately.

### 4.1 Limitations

This study did not include a “sighted” condition for subjects with visual impairment and considered that all subjects in the visually impaired group showed similar impairment. A future study involving this condition may enhance understanding of the results. This study solely used COG velocity to measure postural stability; future studies with additional parameters, including subjects from both sexes and different age groups, will help to further understand postural stability in people with congenital or acquired visual impairment.

## 5 CONCLUSION

The results of this study confirm that subjects with visual impairment, regardless of eyes opening or closure, behave in the same manner as sighted subjects with eyes closed.

## CONFLICT OF INTEREST

None declared.

## AUTHOR CONTRIBUTIONS

Research idea and design were proposed by ZAI and AHA. Review of literature was performed by ZAI and AZA. Data collection and analysis were performed by ZAI, AHA, and AZA. Manuscript preparation and submission were performed as done by ZAI.

## CONSENT TO PARTICIPATE

All subjects were informed about the aims and procedures of the study, and written informed consent was obtained for participation in the study.

## Data Availability

The datasets used in this study are available from the corresponding author on request.
